# Does carbon dioxide pneumoperitoneum enhance wound metastases following laparoscopic abdominal tumor surgery? A meta-analysis of 20 randomized control studies

**DOI:** 10.1007/s13277-014-1812-5

**Published:** 2014-04-18

**Authors:** Xianwei Mo, Yang Yang, Hao Lai, Jun Xiao, Ke He, Jiansi Chen, Yuan Lin

**Affiliations:** 1 Department of Gastrointestinal Surgery, Tumor Hospital of Guangxi Medical University, 22 Shuangyong Road, Nanning, 530021 Guangxi Autonomous Region China; 2 Department of Neck and Head Surgery, Tumor Hospital of Guangxi Medical University, Nanning, 530021 Guangxi Autonomous Region China

**Keywords:** Carbon dioxide pneumoperitoneum, Laparoscopic, Abdominal tumor surgery, Wound recurrence

## Abstract

The mechanisms involved in the development of wound metastasis following laparoscopic abdominal tumor surgery remain unclear. The aim of this study was to accurately assess whether the duration of carbon dioxide pneumoperitoneum (CDP) during laparoscopic abdominal tumor surgery enhances wound metastases. We conducted a systematic review of PubMed, Cochrane Library, and Embase through December 2013 to identify animal experiments comparing wound recurrence between laparoscopic and gasless laparoscopic procedures or open procedures. The outcome of interest was the number of animals with a wound tumor. Meta-regression was used to assess whether heterogeneity was explained by study level covariates (animal model, study size, CDP pressure, duration, and evaluated time). Twenty randomized control studies involving 1,229 animals were included. Wound recurrence was not significant in the laparoscopic surgery (LP) vs. gasless laparoscopic surgery (GLP) subgroups [odds ratio (OR), 2.23; 95 % confidence interval (CI), 0.90–5.55; *P* = 0.08) or the LP vs. laparotomy (LA) subgroups (OR, 0.97; 95 % CI, 0.31–3.00; *P* = 0.08). Overall postoperative wound recurrence results were not significantly different between the study groups and controls (OR, 1.47; 95 % CI, 0.74–2.92; *P* = 0.28). A meta-regression analysis showed that the outcome was not correlated with the covariates (animal model: *P* = 0.82; evaluated time: *P* = 0.30; pressure of CDP: *P* = 0.12; duration time: *P* = 0.80). Current evidence suggests that CDP does not enhance wound metastases following laparoscopic abdominal tumor surgery. Additional large sample, well-designed, randomized, controlled trials are needed to further confirm whether CDP duration in laparoscopic abdominal tumor surgery significantly enhances wound recurrence.

## Introduction

The advantages of laparoscopic surgery, including quicker recovery time, less pain, and shorter hospital stay, are evident in patients with intra-abdominal malignancies [1–4]. However, this technology has limited application for concerns about wound (port-site) metastases following laparoscopic abdominal tumor surgery. Wound metastases following a laparoscopic procedure were first found in a patient with ovarian cancer who underwent abdominoscopy in 1978 [5], and other reports quickly followed. Wound metastases have been found in many types of intra-abdominal malignancies, such as cancers of the genitourinary system, kidney, colon, stomach, and gallbladder during laparoscopic procedures [6–10].

Carbon dioxide pneumoperitoneum (CDP) has been widely used during laparoscopic abdominal surgery to establish optimal visualization of the operative field; however, whether it enhances wound recurrence remains unclear. To date, conflicting results have been published about the effects of CDP on wound metastases. Some randomized control trials (RCTs) have reported that the duration of intraoperative CDP enhances wound metastasis. For example, Hopkins et al. and Mathew et al. regarded CDP as the direct cause of tumor spread and implantation [11, 12], but several other RCTs reported that CDP has no effect on wound recurrence. Mutter et al. suggested that manipulation is the major cause for wound recurrence following laparoscopic abdominal tumor surgery [13], and Gutt et al. suggested that both laparotomy incision and surgical manipulation stimulate local tumor spread more than does CDP [14]. Several reviews have concluded that the etiology for wound metastasis following abdominal tumor surgery is not yet understood [15–17] since a number of factors (i.e., CDP, aerosolization, chimney effect, immune response, and surgical technique) are involved. Thus, whether CDP duration enhances wound metastasis remains unclear. Therefore, it is necessary to reassess all data and elucidate controversial or inconclusive results. We assumed that the duration of CDP enhanced wound recurrence following laparoscopic abdominal tumor surgery and performed a systematic meta-analysis.

## Materials and methods

### Search strategy

The PubMed, Embase, and Cochrane Library electronic databases were used to search for animal studies up to December 2013. The following medical subject heading terms and words were used, in all possible combinations, for the search: “pneumoperitoneum,” “insufflation,” “neoplasm metastasis,” “metastasis,” “recurrence,” “port side,” “trocar side,”“wound,” and “incision”. A filter for identifying comparable studies recommended by the Cochrane Collaboration was used to filter out randomized studies in PubMed and Embase [18]. A manual search of the reference lists of relevant articles was performed. No language or time restrictions were made.

### Eligibility criteria

We included a study in the analysis if it met the following criteria: (1) study design: RCT; (2) population: animals undergoing laparoscopic abdominal tumor procedures; (3) intervention: duration of CDP during the intraoperative period; (4) comparison group: animals undergoing gasless laparoscopic abdominal surgery or animals undergoing open abdominal tumor surgery; and (5) all studies included had to have reported the number of animals with wound tumors. Wound metastasis/recurrence was defined as the number of animals that developed at least one wound with tumor implantation after a laparoscopic or open procedure. We excluded a study if it met any of the following criteria: (1) a design other than RCT; (2) the outcome was not that of the number of animals with a wound tumor; (3) intervention other than CDP duration; and (4) inability to extract the raw data and failure to obtain the data from the authors.

### Data extraction

Data were carefully extracted from all eligible publications by two investigators (Xianwei Mo and Yang Yang) independently, according to the above inclusion criteria. Any disagreements were resolved by discussion with a third reviewer (Yuan Lin) during a consensus meeting. The data extracted included the first author’s last name, publication year, study design, country, animal model, number of animals, tumor cell line, postoperative evaluation date, length of incisions, pneumoperitoneum pressure, and duration.

### Assessment of study quality

We used the method described by Sun et al. to evaluate the quality of the involved studies [19]. The study quality was rated using the following six criteria [20, 21]: (I) peer-reviewed publication (score of 2); (II) random allocation to treatment or control (score of 2); (III) animal species (inbred strain, age-matched, statement of MHC mismatch, score of 2); (IV) sample size (sample sizes of both the control and experimental groups must be clearly defined; score of 1); (V) animal welfare regulations were observed (score of 1); and (VI) statement of potential conflict of interests (funding sources must be clearly stated; score of 1). If information was incomplete for any criterion, half of the corresponding score was assigned. Study quality was stratified into four ranks according to their scores: A (score of 7–9); B (score of 5–6); C (score of 3–4); and D (score of 0–2). Two authors (Xianwei Mo and Yang Yang) evaluated the quality of the studies independently. Discrepancies were resolved by consensus.

### Statistical analysis

Data were analyzed using STATA ver. 12.0 (StataCorp LP, College Station, TX, USA). The statistical analysis for dichotomous variables was performed using the odds ratio (OR) and a random- or fixed-effects model according to the presence or absence of heterogeneity. We used the *Q*-based chi-square test and the *I* ^2^ statistic to assess heterogeneity among studies, with a *P* value <0.10 representing statistical significance. Sensitivity and subgroup analyses were used to explore potential causes of heterogeneity. Subgroup analyses were performed to examine whether the number of animals with wound recurrence varied by CDP duration regardless of whether it was a laparoscopic procedure or another type of surgery (laparoscopic or open surgery). A meta-regression model was used to assess whether heterogeneity was explained by study level covariates (animal model, study size, CDP pressure, duration, and evaluated time).

## Results

### Literature search

**Fig. 1:**
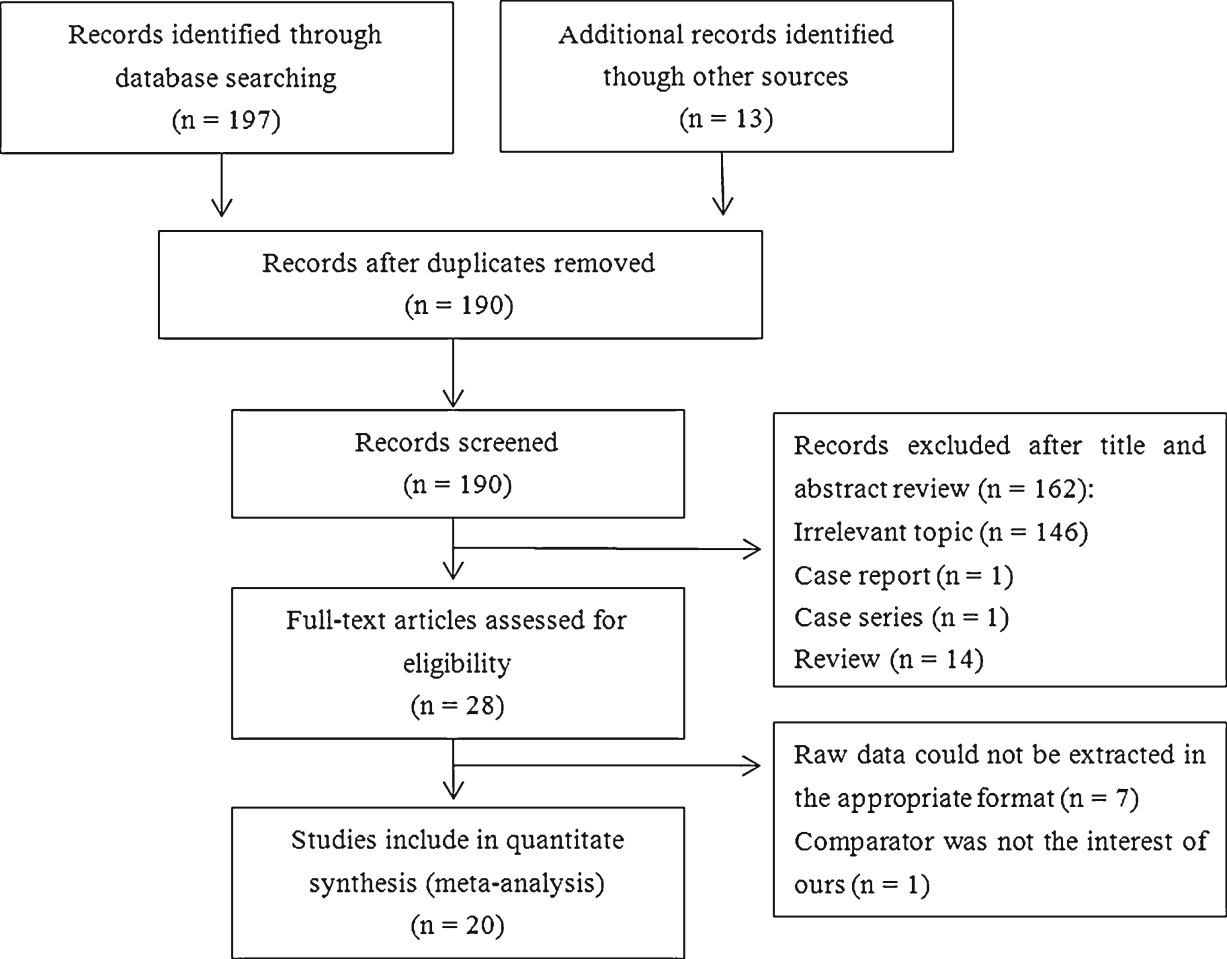
Flow chart for the systematic search and study selection strategy

Figure 1 depicts the PRISMA flow chart for study inclusion and exclusion criteria. A total of 197 records were retrieved from the database search, and 13 records were identified through a manual search of the reference lists of relevant articles. After removing duplicate results, 189 records remained. Of these, 28 studies were selected for full-text examination. Nine were excluded for the following reasons: raw data could not be extracted in the appropriate format (*n* = 7) [12, 22–27], and the comparison group was not of interest (*n* = 1) [28]. Ultimately, 20 studies fulfilled the inclusion criteria for our meta-analysis [11, 14, 29–46].

### Study characteristics

Table 1 shows the characteristics of all studies. The studies were published between 1995 and 2003. The design of all studies was RCT. One study was conducted in Belgium [44], nine in the USA [11, 29–31, 33, 34, 38, 40, 41], four in Australia [32, 36, 37, 46], one in the UK [42], two in France [35, 45], one in Japan [39], one in Germany [14], and one in Israel [43]. A total of 597 animals were included in the study groups and 632 were controls. The most common animal model was the rat [11, 14, 29, 30, 32, 33, 35–37, 42, 44–46], while others included rabbits [39, 41], mice [31, 40, 43], or hamsters [34, 38]. The type of gas used in all studies was carbon dioxide. CDP pressure ranged from 2 to 10 mmHg in the experimental groups and the duration was from 15 min to 2 h. In most of the animal experiments, the sample size was small (<30) [11, 14, 32, 35–37, 39, 41–46], and the follow-up date was short (<4 weeks) [11, 14, 29–32, 36, 37, 39–41, 43, 45, 46]. Eight studies included three group dates and were divided into two groups of comparative dates to allow for a pooled analysis of the outcomes [11, 14, 32, 34, 35, 39, 42, 45]. Fourteen studies described the length of incisions [20, 30, 33, 46–56]. The length of incision was the same in the study and control groups in six studies [20, 48, 52, 53, 55, 57], whereas the length of incision was much shorter in the study group than the control in the other eight studies (Table 1) [30, 33, 46, 47, 50, 51, 54, 56].

**Table 1:** Characteristics of the studies included

First author	Year of publication	Study design	Country	Animal model	Number of animal (case vs. control)	Tumor cell line	Postoperative evaluate date	Length of incisions (case vs. control)	Pressure of CDP	Duration
Jones	1995	RCT	USA	Hamsters	50 vs. 41 (LP vs. GLP)	GW-39 human colon cancer cells	6 weeks	A 1-cm incision + four 5-mm incisions were made in all animals	10 mmHg	10 min
Mathew	1996	RCT	Australia	Rats	12 vs. 12 (LP vs. LA)	Mammary adenocarcinoma tumor cells	1 week	A 2-mm incision vs. a 3-cm incision	2 mmHg	60 min
Mathew	1997	RCT	Australia	Rats	Group 1—12 vs. 12 (LP vs. GLP) Group 2—12 vs. 12 (LP vs. LA)	Viable DA mammary adenocarcinoma cells	6 days	NA	4 mmHg	45 min
Hubens	1996	RCT	Belgium	Rats	10 vs. 10 (LP vs. LA)	Colon carcinoma cell line (CC531)	8 weeks	NA	10 mmHg	15 min
Moine	1998	RCT	France	Rats	Group 1—10 vs. 13 (LP vs. LA) Group 2—10 vs. 13 (LP vs. LA)	Neoplastic cells	8 weeks	NA	12 mmHg	20 min
Wu	1998	RCT	USA	Hamsters	Group 1—32 vs. 31 (LP vs. GLP) Group 2—40 vs. 38 (LP vs. GLP)	GW-39 human colon cancer cell	7 weeks	A 1-cm incision + four 5-mm incisions were made in all animals	10 mmHg	10 min
Watson	1997	RCT	Australia	Rats	12 vs. 12 (LP vs. GLP)	Adenocarcinoma tumor cells	1 weeks	NA	2 mmHg	60 min
Canis	1998	RCT	France	Rats	Group 1—24 vs. 23 (LP vs. LA) Group 2—24 vs. 23 (LP vs. LA)	BD IX rat ovarian adenocarcinoma cells	2 weeks	NA	10 mmHg 4 mmHg	45 min
Lee	1998	RCT	USA	Mice	32 vs. 31 (LP vs. GLP)	Murine colon 26 adenocarcinoma cell line	10 days	Three 5-mm incisions + 1-cm incision were made in all animals	4–6 mmHg	20 min
Lee	2000	RCT	USA	Mice	54 vs. 56 (LP vs. LA)	Murine colon 26 adenocarcinoma cell line	7 days	Three 5-mm incisions + 1-cm incision were made in all animals	4–6 mmHg	20 min
Paik	1998	RCT	USA	Rats	57 vs. 50 (LP vs. GLP)	DHD/K-12 rat colon carcinoma cells	3 weeks	Two 5-mm incisions vs. a 5-cm incision	15 mmHg	NA
Hopkins	1999	RCT	USA	Rats	Group 1—8 vs. 8 (LP vs. GLP) Group 2—8 vs. 8 (LP vs. LA)	Viable MAT B III rat mammary adenocarcinoma cells	7 days	NA	7–8 mmHg	2 h
Hofstetter	2000	RCT	USA	Rats	47 vs. 59 (LP vs. LA)	Colon cancer cells (DHD-K12)	3 weeks	A 3-cm incision vs. a 3-cm incision	10 mmHg	15 min
Tsivian	2000	RCT	Israel	Mice	20 vs. 20 (LP vs. LA)	Renal cell carcinoma cell line	2 weeks	A 3-mm incision vs. a 2-cm incision	5 mmHg	15 min
Gutt	1999	RCT	Germany	Rats	Group 1—11 vs. 11 (LP vs. GLP) Group 2—11 vs. 11 (LP vs. LA)	CC 531 cells	24 days	Three 3-mm incisions vs. three 3-mm incisions three 3 mm incisions vs. a 1 cm incision	7 mmHg	90 min
Ishida	2000	RCT	Japan	Rabbits	Group 1—15 vs. 15 (LP vs. GLP) Group 2—15 vs. 13 (LP vs. LA)	VX2 cancer cells	17 days	Nine 5-mm incisions were made in all animals	8 mmHg	30 min
Tomita	2001	RCT	USA	Rats	50 vs. 52 (LP vs. GLP)	WB2054Ms tumor cells	4 weeks	A 5-mm incision vs. a 4-cm incision	6 mmHg	40.4 min
Wilkinson	2001	RCT	USA	Rabbits	15 vs. 16 (LP vs. LA)	VX-2 carcinoma cell	7–14 days	A 5-mm incision and a 3-mm incision vs. 4-cm incision	8–10 mmHg	NA
Brundell	2002	RCT	Australia	Rats	20 vs. 10 (LP vs. LA)	Dark Agouti mammary adenocarcinoma cells	15 days	A 2-mm incision vs. a 2.5-cm incision	2 mmHg	15 min
Zayyan	2003	RCT	UK	Rats	Group 1—20 vs. 15 (LP vs. GLP) Group 2—20 vs. 15 (LP vs. GLP)	Transplantable rat colon cancer cells	4 weeks	Two 5-mm incisions were made in all animals	4–6 mmHg	30–45 min

*NA* not available, *LP* laparoscopy, *GLP* gasless laparoscopy, *LA* laparotomy, *mm* millimeter, *cm* centimeter

### Quality assessment of the included studies

The quality scores for the studies ranged from 6 to 8, with 10 studies ranked as A [11, 29, 30, 33, 34, 36–38, 45, 46], 10 as B [14, 31, 32, 35, 39–44], and none as C or D (Table 2). The quality was high (A) in half of the studies.

**Table 2:** Quality assessment of the included studies

	Jones et al. [3]	Mathew et al. [46]	Mathew et al. [32]	Hubens et al. [44]	Le Moine et al. [49]	Wu et al. [48]	Watson et al. [36]	Canis et al. [45]	Lee et al. [57]	Lee et al. [53]	Paik et al. [29]	Hopkins et al. [11]	Hofstetter et al. [30]	Tsivian et al. [43]	Gutt et al. [51]	Ishida et al. [52]	Tomita et al. [33]	Wilkinson et al. [41]	Brundell et al. [50]	Zayyan et al. [42]
I	**√**	**√**	**√**	**√**	**√**	**√**	**√**	**√**	**√**	**√**	**√**	**√**	**√**	**√**	**√**	**√**	**√**	**√**	**√**	**√**
II	**√**	**√**	**√**	**√**	**√**	**√**	**√**	**√**	**√**	**√**	**√**	**√**	**√**	**√**	**√**	**√**	**√**	**√**	**√**	**√**
III	**√−**	**√−**	**√−**	**√−**	**√−**	**√−**	**√−**	**√−**	**√−**	**√−**	**√−**	**√−**	**√−**	**√−**	**√−**	**√−**	**√−**	**√−**	**√−**	**√−**
IV	**√**	**√**	**√**	**√**	**√**	**√**	**√**	**√**	**√**	**√**	**√**	**√**	**√**	**√**	**√**	**√**	**√**	**√**	**√**	**√**
V	**√**	**√**	–	–	–	**√**	**√**	**√**	–	–	**√**	**√**	–	–	–	–	**√**	–	**√**	–
VI	–	**√**	–	–	–	–	–	–	–	–	–	–	**√**	–	–	–	–	–	–	–
TS	7	8	6	6	6	7	7	7	6	6	7	7	7	6	6	6	7	6	7	6
G	A	A	B	B	B	A	A	A	B	B	A	A	A	B	B	B	A	B	A	B

√: complete data; – : not stated; √−: incomplete data. I (score of 2): peer-reviewed publication; II (score of 2): Random allocation of treatment and control; III (score of 2): animal species (inbred line, age-matched, MHC (mismatch); IV (score of 1): sample size calculation (sample size of both control and experimental groups must be clarified); V (score of 1): compliance with animal welfare regulation; and VI (score of 1): statement of potential conflict of interest (source of funds must be clarified). Study quality was stratified into four ranks according to their scores: A (a score of 7–9); B (a score of 5–6); C (a score of 3–4); D (a score of 0–2)

*TS* total score, *G* grade

### Quantitative synthesis of data

**Fig. 2:**
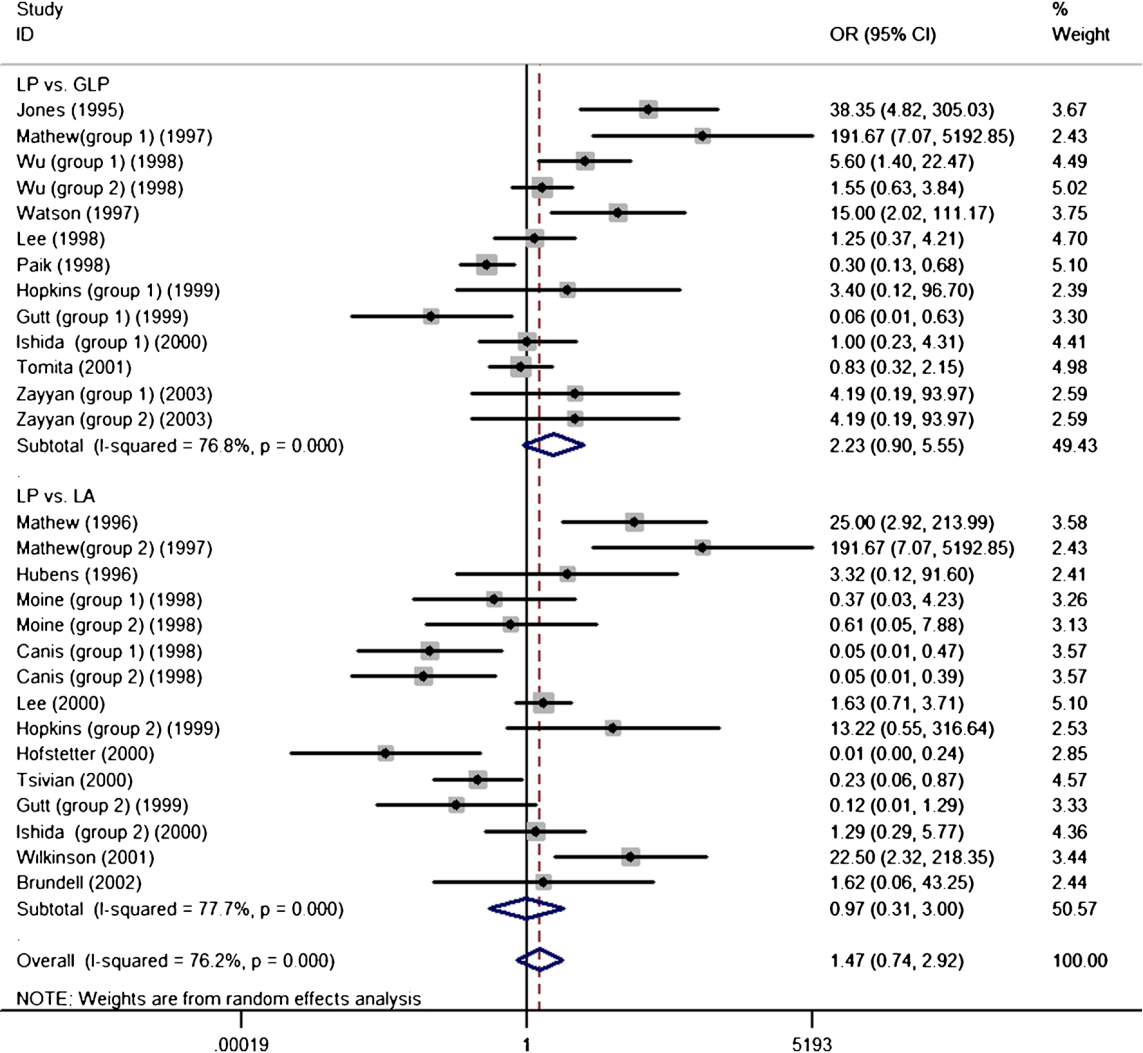
Forest plots of number of animals with wound tumor in subgroup analysis using a random-effect model

Wound recurrence (Fig. 2) was not significant in the laparoscopic surgery (LP) vs. gasless laparoscopic surgery (GLP) subgroups [OR, 2.23; 95 % confidence interval (CI), 0.90–5.55; *P* = 0.08], and the LP vs. laparotomy (LA) subgroups showed consistent results (OR, 0.97; 95 % CI, 0.3–3.00; *P* = 0.08). The overall pooled estimate results showed that wound recurrence was not significant between the study groups and controls (OR, 1.47; 95 % CI, 0.74–2.92; *P* = 0.28). Evidence of significant heterogeneity was seen across trials in the above analysis (*I* ^2^ = 76.2 % and *P*_*Q*_ = 0.00 for heterogeneity). A meta-regression analysis showed that the number of animals with wound tumors was not correlated with the covariates (animal model: *P* = 0.82; evaluated time: *P* = 0.30; CDP pressure: *P* = 0.12; duration: *P* = 0.80).

### Sensitivity analysis

The inclusion criteria of this meta-analysis were subjected to a sensitivity analysis to determine whether modifying inclusion criteria affected the results. A single study involved in the meta-analysis was deleted each time to reflect the influence of each individual dataset on the pooled OR. The corresponding pooled OR was essentially unaltered (data not shown), indicating that our results were statistically sound.

### Risk of publication bias

**Fig. 3:**
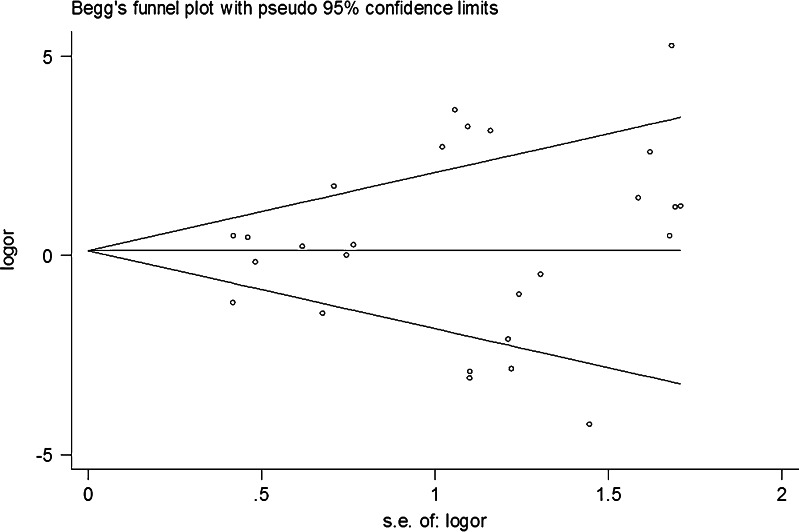
Funnel plot analysis to detect publication bias; *each point* represents a separate study for the indicated association

Publication bias was assessed by Begg’s funnel plots and Egger’s tests. The shapes of the Begg’s funnel plots revealed no obvious asymmetry (Fig. 3). The Egger’s test was then used to statistically assess funnel plot symmetry. The funnel plot was relatively symmetrical, suggesting no publication bias (*t* = 1.16, *P* = 0.26 for the number of animals with a wound tumor). This indicates that the results of these meta-analyses were relatively stable and were unlikely to have been affected by publication bias.

## Discussion

The association between CDP duration during laparoscopic abdominal tumor surgery and wound recurrence is not fully understood. Animal experiments comparing implantation of wound tumors between laparoscopy and laparotomy have presented conflicting results. Several studies have suggested that wound recurrence was not significantly different between laparoscopy and other procedures (gasless laparoscopic surgery or open surgery) [14, 29, 31, 32, 34, 36–43], whereas others have suggested that wound recurrence may have been caused directly by CDP [11, 12, 32]. However, sample size was small in these studies, and they were not individually powered to detect small differences in outcomes. A pooled synthesis of these studies may provide further insight into the results. Our meta-analysis of 20 random control studies provides evidence that CDP duration in laparoscopic abdominal tumor surgery does not enhance wound recurrence.

The underlying mechanism involved in tumor cell wound implantation is uncertain. One possible explanation is trocar use or CDP [11]. Other explanations are the chimney effect, immune response, aerosolization, and poor surgical technique [58]. Thus, the exact mechanism for the development of wound metastases is not yet known, and current evidence shows that CDP is not responsible for these tumors. It seems that the mechanisms are multimodal. Whatever the reason, it is advised that laparoscopic tumor surgery be performed by adequately trained and experienced surgeons and that strict oncologic techniques should be followed.

The interpretation of the results may have been affected by heterogeneity. Large heterogeneity was observed among studies. We performed a sensitivity analysis on these subgroup analyses. A study by Jones et al. with LP vs. GLP subgroups revealed more animals with wound recurrence in the study group than those in the control [38]. Dropping this study did not yield opposite results in the number of animals with wound recurrence, but large heterogeneity remained (*I* ^2^ = 72.4 %). The sensitivity analysis showed that the study by Paik et al. had a large impact on our results [29], as dropping this study yielded opposite findings for the number of animals with wound recurrence (OR, 2.77; 95 % CI, 1.13–6.84, *P* = 0.03). For the LP vs. LA subgroup, more wound recurrence animals were found in the study group than in controls in Mathew et al. (group 2) [32]. Dropping this study did not yield opposite results, and large heterogeneity was still present (*I* ^2^ = 75.7 %). A sensitivity analysis of this subgroup showed that the study by Lee et al. had a large impact on our results [53], and an opposite finding did not occur after dropping this study.

The conflicting results of the studies in our meta-analysis may have been due to (1) the small number of cases in some studies, which increased the possibility that chance accounted for their results; (2) the animal model, CDP pressure, operative time, and tumor cell line in the studies were not uniform, and the follow-up date was short; and (3) several factors, such as trocar placement [39] and peritoneal closure, may have contributed to wound metastasis [59]. These factors in the LP groups were also not uniform.

We included different animal models to evaluate wound recurrence following laparoscopic abdominal tumor surgery and then performed a subgroup analysis based on the variation in wound recurrence with CDP duration, regardless of whether a laparoscopic procedure or another type of surgery (laparoscopic or open surgery) was used. There was sufficient evidence in the 20 randomized control studies to conclude that CDP duration does not enhance wound recurrence following laparoscopic abdominal tumor surgery. This may be the first meta-analysis in which the relationship between CDP duration and wound recurrence has been evaluated systematically. With the accumulating evidence and enlarged sample size, the statistical power to provide more precise and reliable efficiency estimates was enhanced despite the variations in the results from each study. Overall, the published evidence did not support the assumption that CDP duration enhances wound recurrence.

Our meta-analysis had several limitations. First, the animal model, CDP pressure, operative time, and tumor cell lines in the studies were not uniform (Table 1), leading to potential bias in our analysis. Second, several studies had small sample sizes, and follow-up duration was short. It is possible that the lack of a significant difference in the number of animals with port-site tumors between the LP group and other groups was due to these factors. Therefore, large-scale, well-designed trials focusing on different types of animal models are required to establish whether CDP duration enhances wound recurrence. Third, there is no standard tool to evaluate the quality of an animal experiment in a meta-analysis. Jada Queries or Cochrane tools are the most commonly used power tools for this purpose; however, the subjects in those studies were humans. In our meta-analysis, the subjects were animals, so they did not follow the assessment methods in the Jada Queries or Cochrane tools guidelines, such as “double blinding.” We used the method described by Sun et al. to evaluate the study quality [19], which may have produced bias. Four, substantial heterogeneity was present among the studies. Bias was potentially produced by several uncontrolled factors, such as the effects of trocar placement, peritoneal closure, and tumor manipulation [58].

## Conclusions

Current evidence suggests that CDP does not enhance wound metastases following laparoscopic abdominal tumor surgery. Additional large sample, well-designed, randomized, controlled trials are needed to further confirm whether CDP duration during laparoscopic abdominal tumor surgery significantly enhances wound recurrence.
